# Development of a TCR beta repertoire assay for profiling liquid biopsies from NSCLC donors

**DOI:** 10.20517/cdr.2020.07

**Published:** 2020-06-18

**Authors:** Leisa P. Jackson, Benjamin A. Tjoa, Hestia Mellert, Gary A. Pestano

**Affiliations:** ^1^Development, Biodesix Inc., Boulder, CO 80301, USA.; ^2^Cellero, LLC, Bothell, Washington, WA 98021, USA.

**Keywords:** TCRβ, liquid biopsy, Ion Torrent, immunotherapy

## Abstract

**Aim:** The aim of this study was to demonstrate the utility of T-Cell receptor beta (TCRβ) sequencing as a robust method for assessing T-cell repertoire changes in donors with non-small cell lung cancer (NSCLC). We further demonstrated the use of the assay by monitoring repertoire modulation in a defined model antigen system, cytomegalovirus (CMV).

**Methods:** Peripheral blood mononuclear cells from four healthy donors were challenged with a 1-week exposure to whole-cell lysate from CMV-infected cells or CMVpp65_495-503_ peptide (NLVPMVATV). T-cell repertoire perturbations were assessed using the Oncomine TCR Beta-SR Assay and Ion GeneStudio S5 Plus Sequencer. A pp65 tetramer flow cytometry assay was used as an orthogonal method to assess clonal expansion of a subset of CMV-specific T-cells. For evaluation of the assay in peripheral blood lymphocytes from NSCLC donors, five whole blood specimens were evaluated using the same sequencing workflow.

**Results:** The TCR beta assay identified 6,683-61,936 unique clones from 1-2 million reads per sample, and an average of 80% of the total reads were usable for TCR profiling. In the NSCLC donors, TCR convergence and clonality values were consistent with published results and ranged 0.016-0.033 for convergence and 0.09-0.48 for clonality. In the CMV study, TCR sequencing detected the expansion of a common family of clones in all 4 samples in response to antigen stimulation. This expansion corresponded to an increase in pp65 tetramer staining by flow cytometry. Baseline TCR convergence scores ranged 0.009-0.041 and increased 5-fold in one sample as a result of pp65 antigen stimulation.

**Conclusion:** The results of this study demonstrated the utility of profiling of the TCRβ repertoire in a model system and in donors with NSCLC. Additionally, we demonstrated the correlation between RNA-seq methods and protein-tetramer analysis using flow cytometry. These techniques represent an emerging solution that could complement other liquid and tissue diagnostic assays in the clinic and will be of value in predicting host response/resistance and adverse events to immunotherapies. Prospective clinical studies are on-going in which the developed TCR beta assay will undergo further validation.

## Introduction

During infection, and in cancer, the immune system’s response to antigens leads to changes in the T-cell repertoire (TCR). T-cell clonal expansions can be measured by sequencing the antigen-specific loci in the T-cell receptor beta (TCRβ) gene^[1]^. In oncology research, TCRβ sequencing is being explored as a predictor for response and resistance to immunotherapy^[2]^ as well as immune-related adverse events (IRAE) post-immunotherapy^[3]^. Recent studies have focused on two metrics, T-cell clonality and TCR convergence (clones having identical amino acid sequences but variable nucleotide sequences, which can be driven by chronic antigen exposure) as potential biomarkers for predicting response to immunotherapy^[4]^. Non-invasive testing for these markers can be achieved using peripheral blood lymphocytes (PBL).

In this study, whole blood specimens were collected in blood collection tubes (BCT; Streck Inc. NE) from donors previously diagnosed with non-small cell lung cancer (NSCLC)^[5]^. BCT contain preservatives that inhibit cell lysis in the whole blood specimens, thus improving the recovery of intracellular RNA from nucleated cells in whole blood. Specimens were shipped to our laboratory for buffy coat recovery, where RNA was isolated, and specimens were evaluated using TCRβ next-generation sequencing (NGS) on the Ion Torrent GeneStudio S5 Plus sequencer (ThermoFisher Scientific). The Ion Torrent platform was selected to perform this study due to the technology’s high accuracy (low substitution error rate) and rapid turn-around^[4]^. Additionally, to model T-cell repertoire changes after antigen stimulation, primary peripheral blood mononuclear cells (PBMC) were challenged *in vitro* with cytomegalovirus (CMV) antigen using either the single dominant CMV antigenic peptide, pp65_495-503_, or whole-cell lysate prepared from CMV-infected cells. These latter data were intended to validate the utility of the new NGS TCRβ assay by using a defined antigen(s) model system and an established clinical diagnostic technology, flow cytometry. This is one of the first reports demonstrating the correlation between tetramer-based quantification and TCR expression dynamics.

## Methods

### Isolation of PBL RNA from NSCLC donors

Whole blood was collected from 5 donors in Streck BCT®. Specimens were shipped at ambient temperature to our centralized clinical testing laboratory and processed within 4 days after blood was drawn. Whole blood was centrifuged at 800 ×*g* for 20 min, and buffy coat cells were recovered from the plasma/red blood cell (RBC) interface and purified using RBC Lysis Buffer (ammonium chloride-based lysing reagent), according to the manufacturer’s technical data sheet (Tonbo Biosciences, CA). RNA was extracted from purified buffy coat cells using the RNeasy® FFPE Kit, including a DNase treatment step (Qiagen, CA). The specimens used in our study were Institutional Review Board waived as they were remnant, de-identified specimens.

### T-cell expansion with CMV model antigen stimulation

Cryopreserved PBMC, initially recovered by leukapheresis using Ficol, were thawed in X-VIVO^TM^ 15 (Lonza, Basel, Switzerland) medium. We chose 4 different normal healthy, CMV-seropositive, and HLA-A*0201-positive donors for this study. Thawed cells were collected at baseline or cultured for 6 days in the presence of 1 µg/mL CMV whole antigen (partially purified lysate from CMV-infected human fibroblast cells) or 1 µg/mL HLA-A*0201^-^restricted CMV immunodominant peptide (pp65_495-503_, NLVPMVATV). IL-2 (10 U/mL) was added one day after the start of the culture.

For sequencing, cells were lysed using Buffer RLT Plus (Qiagen, CA) supplemented with β-mercaptoethanol and frozen at -80°C until RNA extraction. RNA was isolated according to the manufacturer’s instructions using the RNeasy-Plus® Mini Kit (Qiagen, CA).

PBMC were prepared for flow cytometry using HLA-A*0201-CMVpp65495-503 tetramer-PE reagent (MBL International, MA). Cells were incubated with HLA-A*0201-CMVpp65_495-503_ tetramer-PE reagent (MBL International, Woburn, MA) for 15 min at room temperature, followed by incubation with anti-human CD8a-FITC antibody (clone RPB-T8, BioLegend, San Diego, CA) at 4 °C for 30 min. 7-AAD was included in the final buffer to exclude dead cells. Data were acquired in a FACScan flow cytometer (Becton Dickinson, San Jose, CA) using BD Cell Quest software. Analyses were done using FCS Express version 4 (De Novo Software, Pasadena, CA). Percent HLA-A*0201/CMVpp65_(495-503)_ tetramer-positive population was quantified from live (7-AAD^-^) CD8^+^ cells.

### TCRβ sequencing with Ion Torrent

The Qubit^TM^ RNA HS assay (ThermoFisher Scientific, MA) was used to quantify RNA. Agilent RNA 6000 Nano Assay was used to determine RNA integrity number (RIN) and DV200 scores. cDNA was generated using the SuperScript^TM^ IV VILO^TM^ Kit (ThermoFisher Scientific, MA). Following cDNA synthesis, the input for library preparation was determined based on a functional CD3 RNA qualification assay, which measures the CD3 fraction of mixed cell populations and cDNA amplifiability^[6]^.

**Figure 1:**
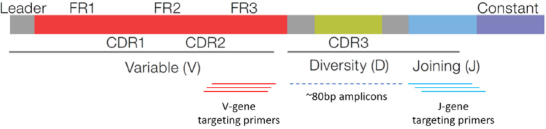
The Oncomine TCR Beta-SR Assay targets the complementarity-determining region 3 (*CDR3*) within the TCR beta chain locus. The assay consists of multiplexed primers that bind to framework region 3 (*FR3*) and joining regions of the locus, producing amplicons approximately 80 bp in length for sequencing. TCR: T-Cell receptor

The Oncomine TCR Beta-SR Assay (ThermoFisher Scientific, MA) was used to sequence *CDR2*, *CDR3*, and joining regions of the TCR Beta locus, as illustrated in Figure 1 (used with permissions from ThermoFisher Scientific). Sequencing was performed on the Ion GeneStudio^TM^ S5 Plus. Twelve specimens were multiplexed per Ion 530^TM^ Chip prior to sequencing using the Ion Chef^TM^ instrument.

## Results

### TCR beta sequencing in NSCLC donor specimens collected in Streck BCT

To assess TCR repertoire features in clinical specimens, whole blood was collected from 5 different NSCLC donors in Streck RNA BCT and shipped to the centralized laboratory. RNA was isolated for TCR sequencing from the buffy coat layer, which contains a large fraction of PBLs. DV200 score, which was developed to measure the quality of highly degraded RNA from formalin-fixed paraffin-embedded (FFPE) samples for RNA-seq, was used to measure RNA quality. This method is suitable for the assessment of PBL RNA from Streck BCT, which tends to be fragmented following shipment at ambient temperature.

The input quantity used for library preparation was based on a functional CD3 RNA qualification assay, which measures both the T-cell RNA content and the amplifiable quality of the sample. As shown in Table 1, the extracted RNA for each sample varied in quality and T-cell content. DV200 values ranged from 52%-77%, and RNA input ranged from 34-400 ng. For donors 1-4, these highly variable inputs were successfully normalized by the qualification assay and resulted in consistent library metrics (1.55-2.13 million raw reads, 73%-83% usable reads, 44,299-61,936 clones). However, for donor 5, the number of raw and usable reads (1.17 million, and 63%, respectively) was relatively low and the assay detected many fewer clones compared to the other samples (*n* = 10,731), potentially due to lower input quantity.

**Table 1:** Sample characterization and TCRβ assay performance metrics for specimens from NSCLC donors

Donor	1	2	3	4	5
DV200 Score	52%	55%	75%	70%	77%
RNA Input (ng)	400	180	72	324	34
Library Conc (pM)	473	457	1101	1004	45
Raw Reads (millions)	1.55	1.99	2.13	1.99	1.17
Productive and Rescued Reads (%)	74	73	79	83	63
Number of Clones	49,914	44,299	58,357	61,936	10,731
Clonality	0.09	0.24	0.15	0.14	0.48
TCR Convergence	0.016	0.021	0.026	0.033	0.005

TCRβ: T-Cell receptor beta; TCR: T-Cell receptor; NSCLC: non-small cell lung cancer

**Figure 2:**
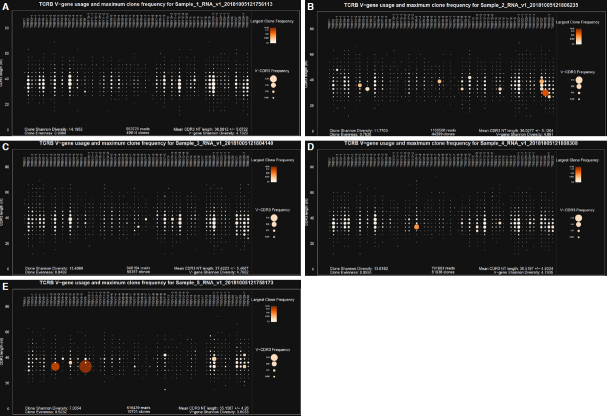
Spectratyping plots for the five NSCLC specimens. Donor 1-5 correspond to A-E, respectively. Circles are bins containing all clones with a particular *V gene-CDR3* nucleotide (nt) length combination. Circle size indicates the frequency of all clones contained in the bin, and color indicates the frequency of the most prevalent clone present in the bin (darker orange with higher frequency). NSCLC: non-small cell lung cancer

For the Oncomine TCR Beta-SR assay, T-cell repertoire metrics are reported using several precalculated outputs, including TCR Convergence and Evenness scores. For this study, Clonality was determined from the Evenness score and was defined as 1- Evenness. Scores ranged 0.005-0.033 for TCR Convergence and 0.09-0.48 for Clonality across the 5 donors [Table 1]. Donor 5 had the highest clonality score, and the expansion of 2 different clones was visible in the Spectratyping plot [Figure 2E, as compared to Figure 2A-D], which is a standard output of the Ion Reporter software.

### T-cell expansion with CMV model antigen stimulation

To study the TCR repertoire changes due to antigen stimulation, T-cell populations from four CMV-seropositive donors were analyzed after a 6-day *in vitro* stimulation with CMV whole antigen (“Lysate”) or a HLA-A*0201^-^restricted CMV immunodominant peptide (“Peptide”).

RNA was isolated from PBMC prior to antigen challenge (“Baseline”) and after stimulation and used to prepare libraries for TCR sequencing following the same workflow used for the NSCLC specimens. As shown in Table 2, a lower input of RNA (25-107 ng) was required due to the high quality of the RNA (RIN Scores 9.9-10) and resulted in similar sequencing metrics to what was seen with relatively fragmented RNA from Streck BCT (1.43-2.16 million raw reads, 81%-85% usable reads).

**Table 2:** Sample characterization and TCRβ assay performance metrics for CMV antigen stimulation study with specimens from normal healthy donors

Donor	Treatment	RIN score	RNA Input (ng)	Library conc (pM)	Raw reads (millions)	Productive and rescued reads (%)	# Clones
1	Baseline	10	46	593	1.6	83	31,804
	Lysate	10	25	788	1.99	85	12,504
	Peptide	10	29	385	1.52	84	26,901
2	Baseline	10	43	708	1.54	82	25,459
	Lysate	9.9	46	1,093	1.99	83	11,250
	Peptide	10	107	687	2.16	83	31,367
3	Baseline	10	38	290	1.45	81	29,964
	Lysate	10	48	630	1.77	84	27,647
	Peptide	10	63	401	1.43	83	26,131
4	Baseline	10	40	563	1.56	83	31,672
	Lysate	10	30	835	1.7	83	6,683
	Peptide	10	29	608	1.78	84	36,051

TCRβ: T-Cell receptor beta; CMV: cytomegalovirus; RIN: RNA integrity number

**Figure 3:**
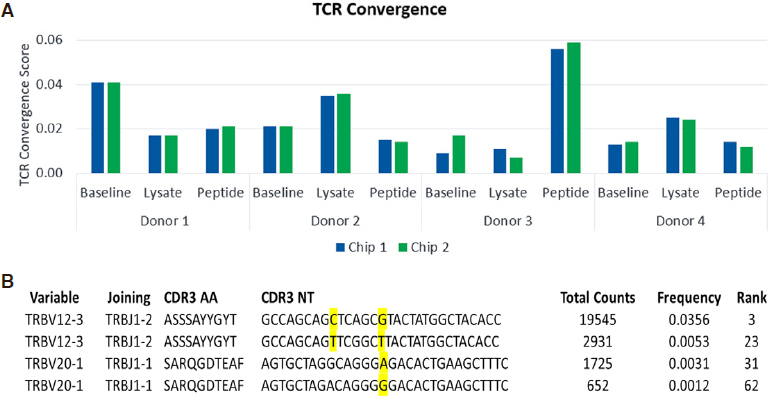
TCRβ convergence scores for the CMV model antigen stimulation study. Results are shown for two replicate Ion 530 chips (A); Two examples of convergent T-cell clones are shown for cells stimulated with pp65 peptide from Donor 3 (B). TCRβ: T-Cell receptor beta; CMV: cytomegalovirus

TCR Convergence scores varied across donors at baseline and in response to antigen challenge, as shown in Figure 3A. The highest TCR Convergence score was observed in peptide-stimulated cells from Donor 3 and was driven by two sets of TCR convergent clones [Figure 3B].

**Figure 4:**
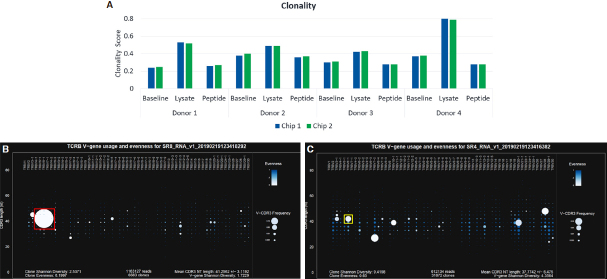
Clonality scores for the CMV model antigen study (A); Spectratyping plots for Donor 4 showing clonal expansion in the lysate condition (B) relative to baseline (C). Color indicates evenness and clonality, where darkest blue is highest evenness and lowest clonality. Size indicates Frequency for each bin containing clones of a particular *V-gene*/CD3 nt length combination, where larger indicates an increasing frequency. TCRβ: T-Cell receptor beta; CMV: cytomegalovirus

Clonality scores increased in the CMV lysate condition relative to baseline for all 4 donors, and the increase was most dramatic for Donor 4 [Figure 4A]. This clonal expansion could be clearly observed in the Spectratyping plots, as shown in Figures 4B and 4C. The most prevalent clone and driver of the high clonality score was observed at 76% frequency within the dataset and is represented by the prominent white circle [Figure 4B, red box]. This clone was present in the corresponding baseline sample at 6.5% frequency [Figure 4C, yellow box].

**Figure 5:**
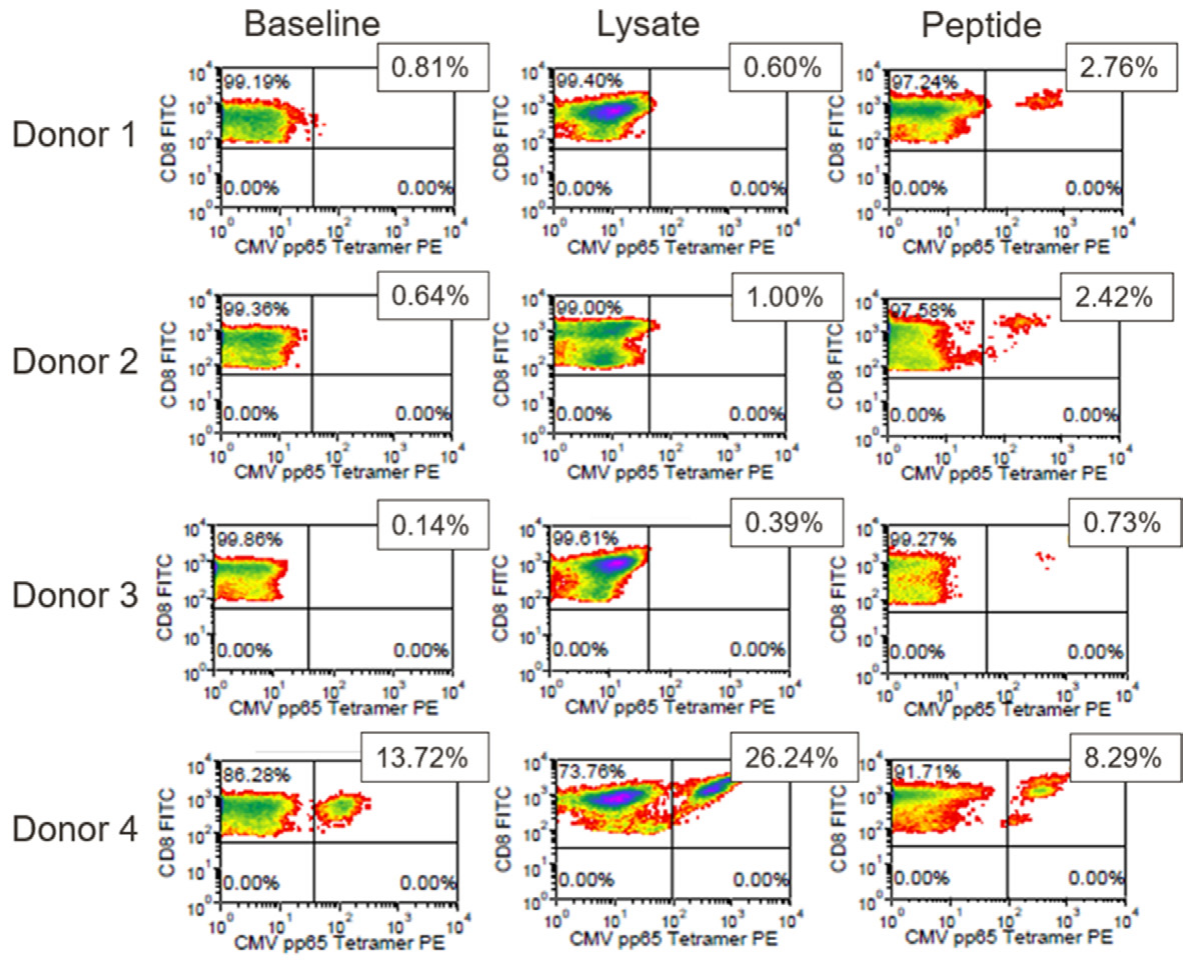
pp65 tetramer assay results for the CMV model antigen study. Positive FITC and PE staining indicates the presence of HLA-A*0201-restricted, pp65(495-503)-responsive CD8+ T-cells. The % positive cells are shown. CMV: cytomegalovirus; FITC: fluorescein isothiocyanate; PE: phycoerythrin

Expansion of CMVpp65_(495-503)_-specific T-cells post-antigen challenge was also analyzed by flow cytometry using HLA-A*0201/CMVpp65_(495-503)_ tetramer reagent. Figure 5 shows CD8^+^ T-cell populations from each of the 4 donors prior to *in vitro* stimulation and after stimulation with CMV lysate or CMVpp65_495-503_ peptide. In the T-cell populations from donors 1-3, a very low abundance of tetramer^+^ CD8^+^ T-cells was observed prior to stimulation, while undetectable or marginal increase in tetramer^+^ CD8^+^ T-cells was observed after stimulation with CMV lysate. However, stimulation of these 3 donors’ T-cells with CMVpp65_(493-503)_ peptide resulted in a significant increase in tetramer^+^ T-cells [Figure 5]. Analysis of the T-cell population from donor 4 showed that 13.72% of the CD8^+^ T cells bound the CMVpp65 tetramer prior to *in vitro* stimulation. The tetramer-positive population expanded to 26.24% after stimulation with CMV whole antigen but decreased to 8.29% after stimulation with CMVpp65_495-503_ peptide.

**Figure 6:**
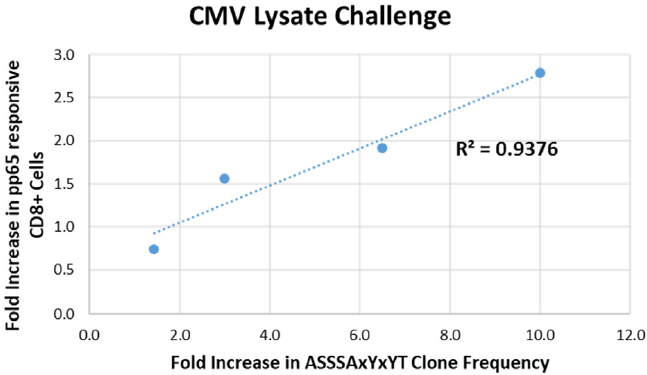
T-cell clone dynamics as measured by the TCR Beta assay correlate with changes in the pp65-responsive CD8+ T-cell population as measured by flow cytometry. The fold change in clone frequency for the ASSSAxYxYT family of clones in response to CMV lysate challenge is plotted relative to the fold increase in % pp65-responsive CD8+ cells in response to CMV lysate challenge for all four donors. Blue dotted line: linear regression analysis. The coefficient of determination (R^2^) for the correlation is shown. TCR: T-Cell receptor; CMV: cytomegalovirus

As shown in Table 3, a single family of clones common to all donors (TRBV12-3 TRBJ1-2 ASSSAxYxYT) expanded in response to stimulation with both pp65 peptide and whole-cell lysate. In the CMV lysate challenge, this expansion observed by sequencing correlated strongly with pp65 tetramer staining [Figure 6]. We compared the fold change in clone frequency for the ASSSAxYxYT family of clones in response to CMV lysate challenge to the % pp65-responsive CD8+ cells in response to CMV lysate challenge for all four donors. The coefficient of determination (R^2^) for the correlation analysis was 0.9376. A similar result was observed in the case of the pp65 peptide stimulation for 3 out of the 4 donors (R^2^ = 0.9454) (data not shown).

**Table 3:** Total reads, frequency, and rank for the TRBV12-3 TRBJ1-2 ASSSAxYxYT family of clones observed across the three treatment conditions for all donors

Donor	Condition	Variable	Joining	CDR3 AA	CDR3 NT	Total counts	Frequency	Rank
1	Baseline	12-3	1-2	ASSSANYGYT	GCCAGCAGTTCGGCGAACTATGGCTACACC	31	0.007%	1300
	Lysate	12-3	1-2	ASSSANYGYT	GCCAGCAGTTCGGCTAACTATGGCTACACC	123	0.010%	470
	Peptide	12-3	1-2	ASSSANYGYT	GCCAGCAGTTCGGCTAACTATGGCTACACC	4003	0.85%	8
2	Baseline	12-3	1-2	ASSSAHYGYT	GCCAGCAGTTCCGCTCACTATGGCTACACC	25	0.004%	2379
	Lysate	12-3	1-2	ASSSAHYGYT	GCCAGCAGTTCCGCTCACTATGGCTACACC	151	0.012%	426
	Peptide	12-3	1-2	ASSSAHYGYT	GCCAGCAGTTCCGCTCACTATGGCTACACC	30031	3.0%,	3
2	Baseline	12-3	1-2	ASSSANYRYT	GCCAGCAGTTCGGCTAACTATCGCTACACC	74	0.012%	639
	Lysate	12-3	1-2	ASSSANYRYT	GCCAGCAGTTCGGCTAACTATCGCTACACC	1528	0.12%	80
	Peptide	12-3	1-2	ASSSANYRYT	GCCAGCAGTTCGGCTAACTATCGCTACACC	150381	15.1%	1
3	Lysate	12-3	1-2	ASSSAYYGYT	GCCAGCAGCTCAGCGTACTATGGCTACACC	96	0.012%	455
	Peptide	12-3	1-2	ASSSAYYGYT	GCCAGCAGCTCAGCGTACTATGGCTACACC	19545	3.6%	3
4	Baseline	12-3	1-2	ASSSANYGYT	GCCAGCAGTTCAGCTAACTATGGCTACACC	489	0.080%	54
	Lysate	12-3	1-2	ASSSANYGYT	GCCAGCAGTTCAGCTAACTATGGCTACACC	6061	0.52%	8
	Peptide	12-3	1-2	ASSSANYGYT	GCCAGCAGTTCAGCTAACTATGGCTACACC	485	0.075%	85

## Discussion

These studies demonstrate that the Oncomine TCRβ assay can detect clonal repertoire features as well as T-cell expansion in response to antigen stimulation with high resolution using PBL isolated from whole blood specimens.

Currently, there is no *in silico* method that can be used to predict the sequence of the T-cell antigen binding domain on the basis of antigen sequence alone. The CMV antigen model system showed that a single family of clones common to all donors (TRBV12-3 TRBJ1-2 ASSSAxYxYT) expanded in response to stimulation with both pp65 peptide and whole cell lysate [Table 3]. Notably, the expansion observed by sequencing correlated with pp65 tetramer staining as shown in Figure 6. Thus, tracking the T-cell repertoire using *CDR3* sequencing has potential clinical utility in the context of monitoring patients following adoptive T-cell therapy in transplants.

Using NSCLC donors, we demonstrated that T-cell profiling can successfully be performed using the buffy coat layer of whole blood collected in cell-free preservative-containing BCT [Table 1, Figure 2]. Preservative blood tubes such as the cell-free DNA and RNA BCT are commonly used in nucleic acid-based liquid biopsy assays in oncology^[7]^. The data in this study may be of value to researchers and clinicians since the amount of blood that can be collected from a patient is often limited, particularly in patients with advanced stage disease. This application provides complimentary biomarker data by providing new diagnostic use the PBL fraction of the same blood tubes already in use for circulating DNA and RNA liquid biopsy applications^[7]^.

Profiling of the TCRβ repertoire using the Ion GeneStudio S5 platform represents a valuable new solution that benefits from the high accuracy (low substitution error rate) of a semiconductor-based sequencing approach; a noteworthy characteristic considering that substitution errors can mimic TCR convergence^[4]^. It is worth noting that the most prevalent clones driving the high convergence score in Donor 3 after exposure to pp65 peptide were not detected at baseline [Table 3]. This suggests that an increase in sample input and read depth for this donor may have been beneficial for detection at baseline. This highlights the importance of sample input/multiplexing as key variables to be optimized during development of a clinical TCRβ assay.

We are currently pursuing studies to further evaluate the clinical utility of sequencing the T-cell immune repertoire in NSCLC patients receiving immunotherapy. An example of one such ongoing study is of NSCLC patients with positive PD-L1 IHC results that are eligible for treatment with immunotherapy^[8]^. We plan to profile the TCR beta repertoire modulation and as well conduct evaluation of circulating RNA for PD-L1 transcripts and IHC expression in tissues with clinical progression. Importantly, this assay is not only promising for the identification of specific T-cell clones in response to therapeutics, but is potentially of utility in prognosing resistance as well as adverse events that may result from immunotherapy^[3]^.

## Declaration

### Copyright

© The Author(s) 2020.
